# Hyperemesis Gravidarum and the Genetics of Growth Differentiation Factor 15 (GDF-15): A Case Report

**DOI:** 10.7759/cureus.115246

**Published:** 2026-08-26

**Authors:** Ana Alfaro, Kailee Myxter, Jackson Thomas, Lacey Vaile-Larson, Jessica E Pullan

**Affiliations:** 1 Research, Rocky Vista University College of Osteopathic Medicine, Ivins, USA; 2 Chemistry and Physics, Southern Utah University, Cedar City, USA

**Keywords:** growth differentiation factor 15 (gdf-15), hg, hyperemesis gravidarum, nausea and vomiting, pregnancy

## Abstract

Hyperemesis gravidarum (HG), the extreme form of nausea and vomiting during pregnancy, significantly impacts the quality of life of a subset of pregnant individuals, leading to hospitalization and severe health complications. Despite the prevalence and intensity of HG, therapeutic approaches remain largely palliative, focusing on symptom management without addressing the underlying pathophysiology. This report delineates a case of severe HG in a 31-year-old woman, highlighting the complexity of diagnosis and management, and discusses the potential of growth differentiation factor 15 (GDF-15) as a therapeutic target. GDF-15, a biomarker linked to placental development and appetite regulation, emerges as a promising avenue for understanding and potentially treating HG. By exploring GDF-15’s role in HG, this case report emphasizes the need for further research into the genetic and biological mechanisms underlying HG. Additional research on HG may facilitate the development of improved management strategies that are both effective for the mother and safe for the fetus.

## Introduction

Nausea and vomiting in pregnancy (NVP), commonly known as morning sickness, is experienced by 70-80% of pregnant women [1]. A subset of 0.3-3.6% of pregnant individuals endure hyperemesis gravidarum (HG), the most severe form of NVP, characterized by debilitating symptoms [1]. HG symptoms include severe nausea and vomiting, an impaired ability to eat and drink normally, and a substantial limitation on daily living activities, ultimately resulting in the principal cause of hospitalization in early pregnancy in the United States [2-4]. While NVP is typically managed with dietary modifications and lifestyle adjustments, HG frequently requires pharmacological intervention, IV hydration, and often hospitalization. Hospitalization criteria for HG patients often include weight loss exceeding 5% of pre-pregnancy weight, dehydration, electrolyte imbalances, and disturbances in acid-base balance [2]. Unfortunately, HG has also been associated with adverse pregnancy outcomes, including low birth weight and preterm delivery [5].

Despite its prevalence, HG research remains limited by confounding factors, restricted access to participants during relevant stages of pregnancy, and the risk-averse nature of pregnancy-related research. The etiology of NVP and HG remains incompletely understood, as current management strategies focus on symptom relief via general antiemetic regimens that are largely ineffective, rather than targeting specific underlying mechanisms [2,6].

Growth differentiation factor 15 (GDF-15), a member of the transforming growth factor-beta superfamily, has emerged over the last decade as a biomarker of interest in HG due to its association with placental development and its elevated levels in the placenta of patients experiencing severe nausea and vomiting during pregnancy [1,7-9]. The focus on GDF-15 is predicated on its hypothesized role in mediating pregnancy-related metabolic adaptations and its potential involvement in the pathophysiology of HG [10]. While the exact mechanism is not discussed in detail here, GDF-15 binds to the GDNF family receptor α-like (GFRAL), activating nausea and vomiting pathways [11]. Previous studies on HG and GDF-15 have investigated the C211G mutation as a potential genetic contributor [12]. However, the following case report challenges the premise that this single mutation is the primary genetic determinant. This suggests that a broader polygenic model may be more appropriate for understanding the drivers of HG.

## Case presentation

A 31-year-old woman with a medical history of type 2 diabetes mellitus presented to the emergency department with hematemesis and severe nausea following a recent sinuplasty and septoplasty. Initial evaluation revealed that the patient was pregnant, with laboratory testing showing elevated beta-human chorionic gonadotropin (β-hCG) levels and the reported last menstrual period indicating approximately 4.5 weeks of gestation (Table 1). The patient initially attributed her symptoms to the postoperative effects of her recent surgeries; however, by eight weeks of gestation, she continued to experience persistent and intractable nausea and vomiting. Despite using ginger and other home remedies, the patient experienced a 4-pound weight loss due to an inability to retain food or liquids. At 12 weeks of gestation, her weight loss had increased to 6 pounds, at which point she was prescribed ondansetron (Zofran) and advised to use Unisom (doxylamine) and vitamin B6. In the context of persistent, severe symptoms, the patient was formally diagnosed with HG at her 12-week appointment. This patient met the Windsor criteria based on symptom onset before 16 weeks of gestation, inability to maintain oral intake, and severe nausea/vomiting with substantial functional impairment; alternative causes were excluded [13]. Pharmacologic therapy was escalated to include metoclopramide and promethazine; however, symptoms continued despite these interventions. Symptoms significantly impaired daily functioning and mental well-being. The patient required multiple hospitalizations for IV hydration and antiemetic therapy, including promethazine and saline at 20 weeks and again at 26 weeks due to continued dehydration and severe nausea.

**Table 1:** Clinical timeline of HG presentation, treatment, and pregnancy outcome HG: hyperemesis gravidarum, β-hCG: beta-human chorionic gonadotropin

Gestational age	Clinical course	Management/outcome
4.5 weeks	Presented to the emergency department with hematemesis and severe nausea following recent sinuplasty and septoplasty. Pregnancy was identified based on elevated β-hCG and last menstrual period.	Symptoms were initially attributed to postoperative effects.
8 weeks	Persistent, intractable nausea and vomiting with inability to retain food or liquids. Weight loss reached 4 lb despite ginger and other home remedies.	Conservative management continued.
12 weeks	Weight loss increased to 6 lb with continued severe nausea and vomiting and substantial functional impairment. Windsor criteria for HG were met, and alternative causes were excluded.	Formally diagnosed with HG. Ondansetron, doxylamine, and vitamin B6 were initiated; therapy was later escalated to metoclopramide and promethazine.
20 weeks	Persistent severe nausea, vomiting, and dehydration despite pharmacologic therapy.	Hospitalized for IV saline hydration and antiemetic treatment, including promethazine.
26 weeks	Recurrent dehydration and severe nausea requiring additional medical intervention.	Hospitalized again for IV hydration and antiemetic therapy.
30 weeks	Symptoms continued to significantly impair daily functioning and ability to maintain full-time employment. Metoclopramide caused substantial sedation.	Metoclopramide was discontinued; patient required medical leave from work.
32 weeks	Symptoms began to improve, and the patient regained weight, surpassing her pre-pregnancy weight.	Continued clinical monitoring.
36 weeks + 6 days	Spontaneous rupture of membranes occurred.	Uncomplicated vaginal delivery with favorable maternal and fetal outcomes.

By 30 weeks gestation, the patient reported significant sedation from metoclopramide, necessitating its discontinuation. The persistent severity of symptoms, including dehydration and weight loss, impaired her ability to maintain full-time employment, leading to medical leave. By the third trimester, however, the patient began to regain weight and surpassed her pre-pregnancy weight by 32 weeks. At 36 weeks and six days, the patient experienced spontaneous rupture of membranes, and subsequent vaginal delivery proceeded without complication. Despite the clinical course, maternal and fetal outcomes were favorable, with the neonate weighing 6 lb 6 oz and having an Apgar score of 8.

**Figure 1:**
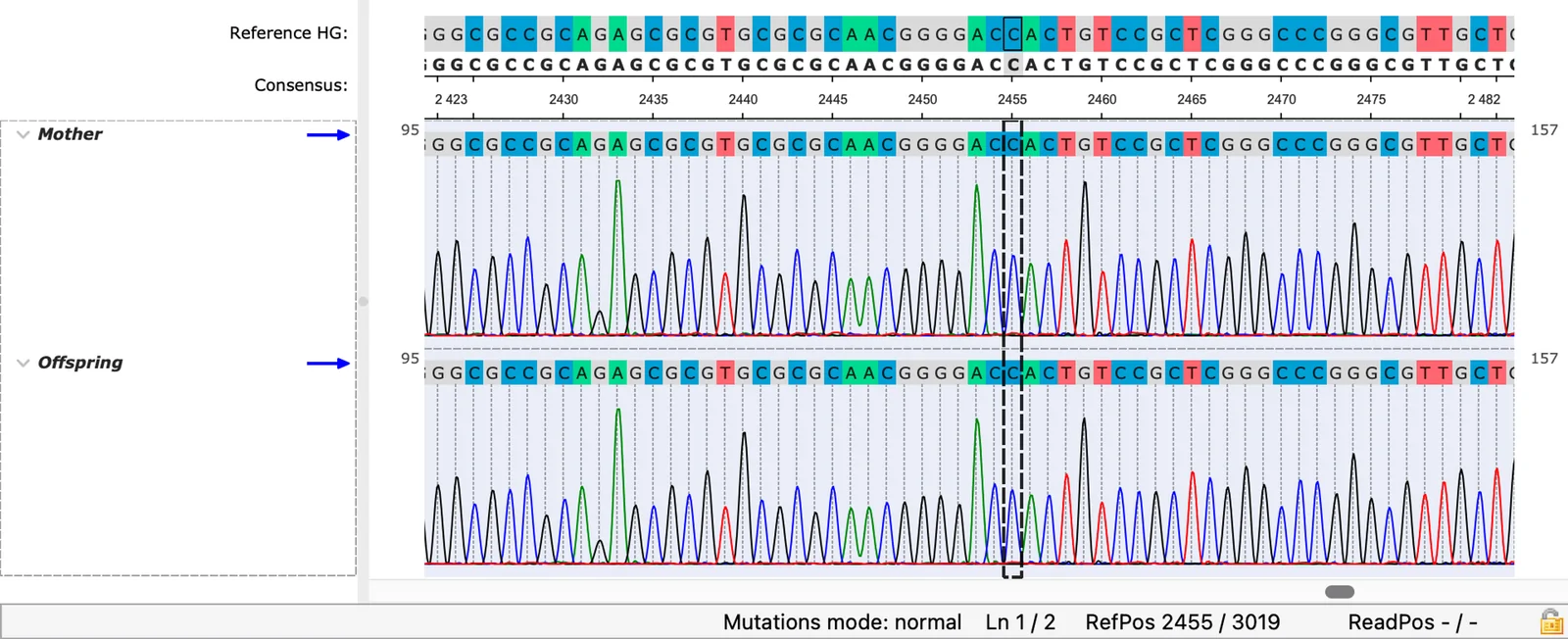
Comparative analysis of GDF-15 reference sequence to mother and offspring The GDF-15 reference sequence is shown at the top, with the maternal sequence in the middle and the offspring sequence at the bottom. The dashed box indicates the genomic position of the reported variant. Sequence alignment demonstrated no nucleotide change at this position in either the mother or offspring relative to the reference sequence. GDF-15: growth differentiation factor 15

To explore the genetic basis of HG, DNA samples were collected from both the mother with HG and her offspring, with a focus on sequencing the C211G mutation in the GDF-15 gene, which is hypothesized to contribute to the severity of her HG symptoms [12]. Serum samples for GDF-15 measurement were unavailable; therefore, buccal swabs were used to obtain genetic material. Genetic analysis was performed on both the mother and her offspring; sequencing revealed that both were homozygous for the wild-type allele (Figure 1). The homozygous wild-type genotype in both the mother and offspring does not support the C211G mutation causing HG in this case, as indicated by the dashed box in Figure 1.

PCR sequencing

Cotton buccal swab samples were collected from the mother diagnosed with HG and her offspring. Genomic DNA was extracted using a Link Genomic DNA Mini Kit (Thermo Fisher Scientific Inc., Waltham, MA, USA) according to the manufacturer’s protocol to obtain high-purity DNA suitable for sequencing. Nuclease-free water was substituted for the elution buffer in preparation for Sanger sequencing because ethylenediaminetetraacetic acid (contained in the elution buffer) can interfere with Sanger sequencing.

Polymerase chain reaction (PCR) was used to amplify key regions of the GDF-15 gene. Primers described by Fejzo et al. [9] were used and validated [9]. The forward primer is GDF15_FORWARD: 5′-CAGCTCAGCCTTGCAAGAC-3′ and the reverse primer is GDF15_REVERSE: 5-′GGATTGTAGCTGGCGGGC-3′. Each 50 µL PCR reaction contained 1X GoTaq Hot Start Green Master Mix (Fisher Scientific, REFM512B), template DNA at an approximate final concentration of 10 ng/µL, and forward and reverse primers each at a final concentration of 0.2 µM. The reaction was adjusted to volume with nuclease-free water. PCR was carried out using a SimpliAmp Thermal Cycler (2280019116106; Thermo Fisher Scientific Inc., Waltham, MA, USA) under optimized cycling conditions, with an annealing temperature of 62°C.

After PCR, the product was visualized using a 1.5% agarose gel containing ethidium bromide at a final concentration of 0.6 µg/mL. For visualization, 6 µL of PCR product was loaded into each well and compared with a well containing 3 µL of 1 kb DNA ladder (Invitrogen), 2 µL of deionized water, and 1 µL of 6X DNA loading dye. Electrophoresis was run at 100 V for approximately 80 minutes using an OWL Easycast B1 (Thermo Fisher Scientific Inc., Waltham, MA, USA). The expected product size was approximately 400 base pairs.

Sanger sequencing was performed by the University of Nevada, Reno Genomic Services. Samples (10 µL of PCR product and 2 µM of primer stocks for each sample) were dried down in a Speed-Vac. PCR was performed using BigDye Terminator v3.1 (Thermo Fisher Scientific Inc., Waltham, MA, USA) in a 10 µL reaction. PCR products were purified using BigDye Xterminator (Thermo Fisher Scientific Inc., Waltham, MA, USA) and analyzed by capillary electrophoresis (SeqStudio, Thermo Fisher Scientific Inc., Waltham, MA, USA).

Sequence alignment and variant analysis were performed using ApE and NCBI BLAST. Sample sequences were submitted to NCBI BLAST using the human genome as a reference. Following BLAST analysis, the alignment was examined for mutations at the target locus by identifying the surrounding sequence. The analysis specifically targeted the variant rs372120002, a T-to-G substitution at position 211 that results in a cysteine-to-glycine amino acid change. Chromatogram peaks were examined in ApE to determine zygosity, where single peaks represented homozygosity and dual peaks indicated heterozygosity. Sequences from Sample 1A (maternal) and Sample 1B (offspring) were compared to assess potential variation in the GDF-15 gene (corresponding .ab1 files to view chromatograms are available upon request).

## Discussion

While the patient in this case was managed with standard antiemetics, the persistent severity of her symptoms, including recurrent hospital visits and significant health risks, highlights the urgent need for mechanism-based therapies such as those discussed below.

The data from Mares et al.’s study further demonstrate the reliance on multiple medications and the lack of therapies targeting underlying mechanisms [2]. Over half of the patients surveyed reported needing three or more medications to manage symptoms, highlighting the difficulty in achieving effective relief. Moreover, 68% of patients experienced notable side effects, from fatigue and constipation to depression and anxiety, revealing the double burden of treatment and disease [2]. Despite taking upwards of seven medications, symptoms were persistent, and medication-related adverse effects impaired her ability to work and disrupted her mental health [2]. This therapeutic inefficacy may stem from the fact that current treatments, such as ondansetron and doxylamine, are aimed solely at symptom control via the chemoreceptor trigger zone and do not address biological drivers such as GDF-15 [14,15]. As GDF-15 has been implicated in the pathophysiology of HG, the lack of therapies that modulate this pathway suggests a critical gap in our treatment strategy; however, the C211G mutation does not appear to be the sole cause of this condition [9,16,17].

Given the absence of the c.211G>C variant in both the maternal and offspring DNA, the analysis suggests that this mutation did not contribute to this patient's HG symptoms. However, the limited scope of the single-locus investigation contrasts with the current literature, which considers the polygenic nature of HG in populations abroad [12,17]. Therefore, whole-genome sequencing studies of individuals with HG at different stages of pregnancy in the United States may help elucidate broader genetic contributions to HG and the effects that other mutations may have on it.

Therapeutic strategies under investigation largely focus on disrupting GDF-15-GFRAL signaling through anti-GFRAL antibodies, anti-GDF-15 antibodies, or ligand traps. Although no therapies are currently approved for HG and pregnancy safety remains uncertain, a phase II clinical trial evaluating an antibody-based approach has reported encouraging preliminary findings [15,18]. Future studies should also examine therapeutic insights from other nausea- and vomiting-associated conditions, including weight-loss treatment and cancer cachexia, potentially through GLP-1 receptor antagonists such as avexitide [19]. Additional investigation into altered fuel utilization, amino acid catabolism, and skeletal muscle loss may reveal new therapeutic targets and further clarify the pathophysiology of HG [17,20].

A complementary strategy involves identifying patients at elevated genetic risk and initiating treatment before severe symptoms develop. Genetic screening could identify individuals with variants associated with altered GDF-15 expression or heightened sensitivity to GDF-15 during pregnancy. For these patients, early, targeted modulation of the GDF-15 pathway may provide more effective symptom control while minimizing fetal risk [15,16]. This personalized approach could support earlier intervention, improve maternal and fetal outcomes, and represent a major shift in the management of HG.

## Conclusions

The report’s primary finding is that despite the patient’s severe HG symptoms, neither she nor her offspring carried the C211G mutation. Both were homozygous for the wild-type allele. As this case does not support the C211G mutation as the primary driver of HG, larger population-based studies are required to confirm the polygenic nature of the condition. Additionally, sequencing of the entire GDF-15 gene and other HG-associated genes is necessary to understand other genetic drivers.
